# Stieleriacines, *N*-Acyl Dehydrotyrosines From the Marine Planctomycete *Stieleria neptunia* sp. nov.

**DOI:** 10.3389/fmicb.2020.01408

**Published:** 2020-07-16

**Authors:** Birthe Sandargo, Olga Jeske, Christian Boedeker, Sandra Wiegand, Jan-Peer Wennrich, Nicolai Kallscheuer, Mareike Jogler, Manfred Rohde, Christian Jogler, Frank Surup

**Affiliations:** ^1^Department of Microbial Drugs, Helmholtz Centre for Infection Research (HZI), Braunschweig, Germany; ^2^German Centre for Infection Research (DZIF), Partner Site Hannover-Braunschweig, Braunschweig, Germany; ^3^Leibniz Institute DSMZ – Deutsche Sammlung von Mikroorganismen und Zellkulturen, Braunschweig, Germany; ^4^Department of Microbiology, Radboud University, Nijmegen, Netherlands; ^5^Department of Microbial Interactions, Institute of Microbiology, Friedrich Schiller University Jena, Jena, Germany; ^6^Central Facility for Microscopy, Helmholtz Centre for Infection Research (HZI), Braunschweig, Germany

**Keywords:** planctomycetes, secondary metabolites, biofilm, antimicrobial activity, dehydroamino acids, signaling

## Abstract

Bacteria of the phylum *Planctomycetes* occur ubiquitously in marine environments and play important roles in the marine nitrogen- and carbon cycle, for example as scavengers after phototrophic blooms. Here, we describe the isolation and characterization of the planctomycetal strain Enr13^T^ isolated from a *Posidonia* sp. biofilm obtained from seawater sediment close to Panarea Island, Italy. Phylogenetic tree reconstruction based on 16S rRNA gene sequences and multi-locus sequence analysis supports the delineation of strain Enr13^T^ from characterized species part of the phylum of *Planctomycetes*. HPLC-MS analysis of culture broth obtained from strain Enr13^T^ revealed the presence of lipophilic metabolites, of which the major compound was isolated by preparative reversed-phase HPLC. The structure of this compound, named stieleriacine D (**1**), was elucidated utilizing HRESIMS, 1D- and 2D-NMR data as a new *N*-acylated dehydrotyrosine derivative. Its biosynthesis was proposed based on an *in silico* gene cluster analysis. Through analysis of the MS/MS spectrum of **1** and its minor derivative, stieleriacine E (**2**), it was possible to assign the structure of **2** without isolation. **1** showed antibacterial activity, however, the wide distribution of structurally related compounds indicates a potential role as a signaling molecule.

## Graphical Abstract

Stieleriacine D, a secondary metabolite identified in the supernatant of cultures of strain Enr13^T^.

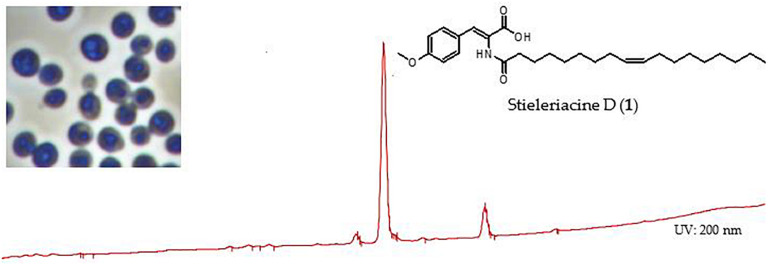

## Introduction

The bacterial phylum *Planctomycetes*, along with *Chlamydiae*, *Verrucomicrobia* and other sister phyla, belongs to the PVC superphylum, which has medical and biotechnological relevance (Wiegand et al., 2018). Although Planctomycetes have a cell envelope architecture similar to that of Gram-negative bacteria (Jeske et al., 2015; van Teeseling et al., 2015), certain aspects of their cell biology are unusual. Members of the class *Planctomycetia*, currently the largest class within the phylum *Planctomycetes*, divide through budding, while binary fission was observed as cell division mode in the class *Phycisphaerae*. All characterized Planctomycetes lack “canonical” divisome proteins including the otherwise universal FtsZ (Jogler et al., 2012; Wiegand et al., 2020). Initially, Planctomycetes were suggested to be beyond the bacterial cell plan, but with the advent of novel microscopic techniques and development of genetic tools for Planctomycetes (Jogler et al., 2011; Rivas-Marin et al., 2016), several eukaryote-like chracteristics of Planctomycetes have been reassessed. Proposed intracellular compartments turned out to be rather invaginations of the cytoplasmic membrane (Boedeker et al., 2017), and the cell envelope of Planctomycetes was reinterpreted as similar to that of Gram-negative bacteria (Devos, 2014).

Some Planctomycetes appear as free-living cells in soil, freshwater, or marine habitats, in which these heterotrophs play an important role in nitrogen fixation (Delmont et al., 2018). Others live associated with eukaryotic organisms, such as sponges and diatoms, cyanobacteria and micro- or macro-algae, frequently forming biofilms (Faria et al., 2018). Their dominance on algal surfaces is surprising given their slow growth compared with other natural competitors in this ecological niche, such as members of the *Roseobacter* clade (Frank et al., 2014; Wiegand et al., 2020). Thus, members of the phylum *Planctomycetes* are suspected to produce small molecules, possibly to compensate for the growth disadvantage in microbial communities on biotic surfaces (Lage and Bondoso, 2014).

In addition to their interesting cell biology, *Planctomycetes* is the bacterial phylum with the highest numbers of predicted genes of unknown function (40–55% of the annotated proteins) (Overmann et al., 2017). Employing comparative genomics, it was shown that the compartmentalization and complex life cycle of Planctomycetes are associated with a sophisticated signal transduction repertoire (Jogler et al., 2012). Their large genomes of up to 12.4 Mb contain a multitude of gene clusters putatively involved in secondary metabolite production (Jeske et al., 2013; Ravin et al., 2018; Yadav et al., 2018). While extracts of various Planctomycetes showed antimicrobial activity (Graça et al., 2016; Jeske et al., 2016) and anticancer effects (Calisto et al., 2019), only very recently the first structures of secondary metabolites from Planctomycetes have been elucidated (Panter et al., 2019; Kallscheuer et al., 2020).

In comparison to well-investigated phyla, the phylum *Planctomycetes* is still under-sampled (Wiegand et al., 2020), thus, we are continuing our sampling approach for new strains belonging to the phylum. We herein present the characterization of strain Enr13^T^ isolated from *Posidonia* leaves close to a gas escape in 20 m water depth close to the island Panarea, Italy, and identified the structure of two new secondary metabolites it produces.

## Results and Discussion

### A Novel Species of the Genus *Stieleria*

#### Isolation of Strain Enr13^T^ and Phylogenetic Analysis

**FIGURE 1:**
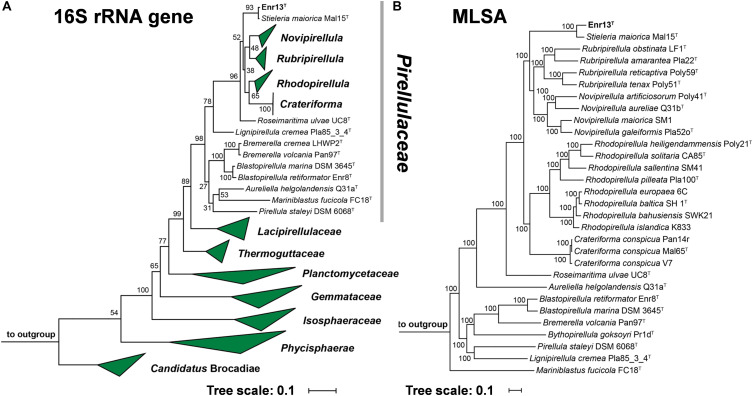
**(A)** 16S rRNA gene sequence- and MLSA-based phylogenetic trees showing the position of strain Enr13^T^. Phylogeny was computed using the maximum likelihood method. Bootstrap values after 1000 re-samplings (16S rRNA gene)/500 re-samplings (MLSA) are given at the nodes (in %). **(B)** The outgroup of the 16S rRNA gene sequence-based tree consists of three 16S rRNA genes from species outside of the phylum *Planctomycetes*, but part of the PVC superphylum. Two species of the family *Planctomycetaceae* (*Planctopirus limnophila* and *Gimesia maris*) served as outgroup in the MLSA-based tree.

Leaves of *Posidonia* sp. were collected close to the island Panarea, Italy, for the targeted isolation of novel Planctomycetes from biotic surfaces. Since all known members of the phylum *Planctomycetes* show high tolerance against β-lactam antibiotics, likely as a result of presence of β-lactamase enzymes (Godinho et al., 2019), compounds of this class were used to direct selection. Strain Enr13^T^ was initially identified as a Planctomycete by partial 16S rRNA gene sequencing and was then subjected to full genome sequencing. 16S rRNA gene sequence comparison and multi-locus sequence analysis (MLSA, Figure 1) revealed that strain Enr13^T^ clusters within the family *Pirellulaceae*. The closest relative of strain Enr13^T^ is *Stieleria maiorica* Mal15^T^, with a 16S rRNA sequence identity of 99.5% (Supplementary Table S2). Based on the proposed threshold value for delineation of species of 98.7% 16S rRNA gene similarity (Stackebrandt and Ebers, 2006), the novel strain would not represent a novel species. We already observed earlier that closely related Planctomycetes can very well belong to separate species, despite having 16S rRNA sequence identities above the threshold value (Kohn et al., 2020). Thus, we evaluated additional markers for analysis of the phylogenetic position of strain Enr13^T^, such as average nucleotide identity (ANI) and identity of a partial sequence of the *rpoB* gene coding for the β-subunit of RNA polymerase (Bondoso et al., 2013; Kim et al., 2014). With 90.8% (Supplementary Table S2) sequence identity of the partial *rpoB* sequence between strain Enr13^T^ and *S. maiorica* Mal15^T^, the *rpoB* marker supports the classification as two separate species, given that an identity of <96.3% is required to clearly differentiate between species (Bondoso et al., 2013). Concordantly, the ANI value is as small as 80.1% (Supplementary Table S2), with the species threshold usually seen at 95–96% (Kim et al., 2014). An average amino acid identity (AAI) of 81.3% and percentage of conserved proteins (POCP) of 72.8% between strains Mal15^T^ and Enr13^T^ clearly indicate the allocation to the same genus (Konstantinidis and Tiedje, 2005; Qin et al., 2014). In accordance with the above-mentioned values, we propose that strain Enr13^T^ forms a new species within the genus *Stieleria*.

#### Morphological Characterization and Genome Information

**FIGURE 2:**
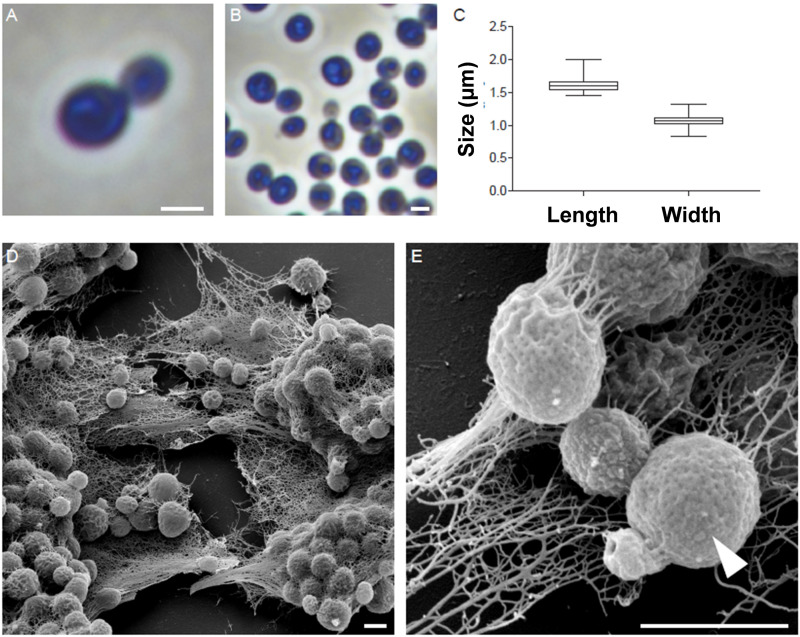
Morphology of strain Enr13^T^. Representative phase-contrast and scanning electron microscopic (SEM) micrographs of strain Enr13^T^. Round to ovoid cells divide by polar budding **(A)**. Phase-contrast micrographs show a high variation of structures within the cells **(B)**. Average cell size (*n* = 500): 1.6 ± 0.1 μm in length and 1.1 ± 0.1 μm in width **(C)**. Cells form dense biofilms with high amounts of exopolysaccharides **(D)**. Individual cells are surrounded by a wrinkled layer or show crateriform structures (arrow head) on their surface **(E)**. Scale bar: 1 μm.

The morphology of strain Enr13^T^ was assessed using light and scanning electron microscopy (Figure 2). Cells appear round to ovoid and divide by polar budding (Figure 2A). Phase-contrast micrographs show a high structural variation among the cells (Figure 2B), and individual cells are usually surrounded by a wrinkled layer or show crateriform structures (Figure 2E). The average cell is 1.6 ± 0.1 μm in length and 1.1 ± 0.1 μm in width (Figure 2C) and forms pink-colored colonies on solid media. Cells of strain Enr13^T^ have a similar shape as cells of *S. maiorica* Mal15^T^, but are slightly smaller (Mal15^T^: 1.9 × 1.4 μm). Both species do not differ in colony pigmentation. During the exponential growth phase in liquid culture, Enr13^T^ cells are motile swimmers, and, once sessile, start forming dense biofilms with high amounts of exopolysaccharides (Figure 2D).

The closed genome of strain Enr13^T^ has a size of 10,975,817 base pairs and a G+C content of 58.9% (Supplementary Table S1). With this size, the genome was the largest recently published in a study targeting 89 planctomycetal genomes (Wiegand et al., 2020). Only *Fimbriiglobus ruber* SP5^T^ features a slightly larger chromosome (Ravin et al., 2018). The genome of strain Enr13^T^ is about 1.1 Mb larger than that of its closest relative, *S. maiorica* Mal15^T^. It carries 7,797 protein-coding genes, 12% more than *S. maiorica*. Interestingly, the rate of hypothetical proteins is only 2% higher, implying that the high count of proteins comes with additional functionalities. As strain Enr13^T^ features significantly more transposable features than *S. maiorica*, these elements might be directly related to the elevated genome size.

#### Physiological Characterization

Strain Enr13^T^ is an aerobic heterotroph and reached a maximal growth rate of 0.054 h^–1^ (division time of 13 h) during laboratory-scale growth experiments in M1H NAG ASW medium, which is 40% lower compared to *Stieleria maiorica* Mal15^T^ (0.093 h^–1^). At constant agitation and optimal growth conditions at pH 7.5 and 28°C (Supplementary Figure S1), strain Enr13^T^ reached the stationary phase after approximately 60–70 h (initial OD_600_ of 0.05, final OD_600_ of 0.6). Cultivation experiments for determination of pH and temperature optima confirmed a mesophilic growth profile from 9°C to at least 35°C (Supplementary Figure S1A) and demonstrated the ability to grow between pH 6.5 and 9.0 (Supplementary Figure S1B). The temperature optimum differs significantly from the optimal growth temperature of *S. maiorica* Mal15^T^ of 35°C, while the pH optimum of both strains is identical. Additional assays demonstrated that strain Enr13^T^ is catalase-negative and oxidase-positive. As described previously for other Planctomycetes (Wiegand et al., 2020), a conventional Gram-staining was also not possible in case of strain Enr13^T^.

#### Strain Description

Based on the phylogenetic analysis and the physiological and morphological differences compared to *S. maiorica*, we propose strain Enr13^T^ as a member of a new species within the genus *Stieleria* of the family *Pirellulaceae*, and propose the name *Stieleria neptunia* sp. nov. with Enr13^T^ as the type strain.

Description of *Stieleria neptunia* sp. nov.

nep.tu’ni.a. N.L. fem. adj. *neptunia* of Neptune; corresponding to the origin of the strain from the Neptune grass *Posidonia* sp.

Cells are round to ovoid (length: 1.6 ± 0.1 μm, width: 1.1 ± 0.1 μm), form aggregates and divide by polar budding. Cells show crateriform structures, whereas a stalk or holdfast structure was not observed. Cells grow over ranges of 9–35°C (optimum 28°C) and pH 6.5–9.0 (optimum 7.5). Colonies are pink. The genome of the type strain has a G+C content of 58.9% and a size of 10.98 Mb. The type strain is Enr13^T^ (DSM 100295^T^ = LMG 29144^T^) isolated from *Posidonia* sp. leaves sampled close to Panarea island, Italy.

### Novel Secondary Metabolites Isolated From Strain Enr13^T^

#### Secondary Metabolite Screening

To examine the presence of secondary metabolites, ethyl acetate extracts of strain Enr13^T^ cultures were analyzed by HPLC-MS coupled with DAD/UV-Vis spectroscopy (Jeske et al., 2016). A peak corresponding to the molecular ion cluster at *m/z* 458.4 in positive mode and 456.3 in negative mode was detected in the lipophilic area of the chromatogram, accompanied by a minor derivative at *m/z* 460.4 in positive mode and 458.3 in negative mode. These data indicate the presence of metabolites with molecular weights of 457 and 459 Da, respectively.

#### Isolation and Structure Elucidation

**FIGURE 3:**
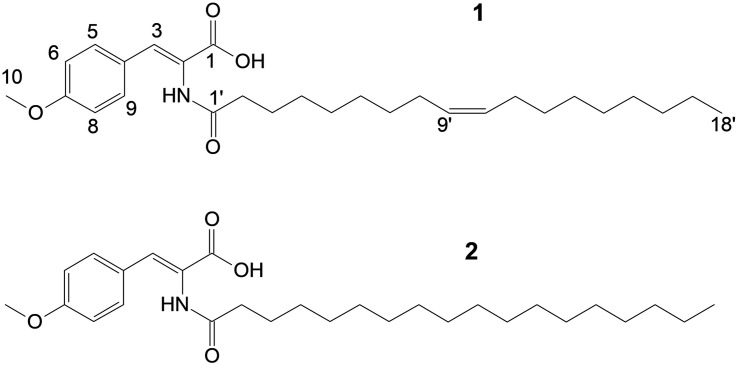
Structures of stieleriacine D **(1)** and E **(2)**.

Utilizing preparative HPLC, the major metabolite **1** was isolated from the fermentation broth of strain Enr13^T^ as an off-white, amorphous solid (1.0 mg). The molecular formula C_28_H_43_NO_4_ of **1** was determined based on the [M+H]^+^ peak at *m/z* 458.3282 in the HRESIMS spectrum, implying eight degrees of unsaturation. ^1^H- and HSQC- (Heteronuclear Single Quantum Coherence) NMR spectra led to the identification of two aromatic methines (δ_C/H_ 113.9/6.93, CH-6/8; 131.5/7.57, CH-5/9; each integrating to two), and three sp^2^ hybridized methines (δ_C/H_ 129.60/5.32, CH-10′; 129.63/5.32, CH-9′; 131.5/7.21, CH-3) (Figure 3 and Table 1). Furthermore, two singlets could be attributed to an oxygenated methyl group (δ_C/H_ 55.2/3.77, CH_3_–10), as well as an amide proton (δ_H_ 9.26, 2–NH), and two triplets to a methylene (δ_C/H_ 33.6/2.18, CH_2_–2′), and a methyl group (δ_C/H_ 13.9/0.85, CH_3_–18). Multiplets at δ_H_ 1.98, integrating to two, and δ_H_ 1.47 were assigned to the methylenes δ_C_ 26.5 (C-8′ and C-11′) and δ_C_ 24.5 (C-3′) and a large multiplet at δ_H_ 1.26 belonging to ten methylenes (δ_C_ 22.1, 31.2, 8 × 28.5-29.0, CH_2_–4′-7′ and CH_2_–12′-17′). Furthermore, the ^13^C-NMR data revealed one carboxylic acid (δ_C_ 166.6, C–1), one carboxamide (δ_C_ 174.5, C–1′), one sp-hybridized carbon (δ_C_ 125.1, C–2), two aromatic quaternary carbons (δ_C_ 126.3, C–4), of which one is oxygenated (δ_C_ 159.9, C–7). HMBC (Heteronuclear Multiple Bond Correlation) correlations from 3 –H to C–1, C–5/C–9, from 5/9–H to C–3, C–7, from 6/8–H to C–4, C–7, from 10–H_3_ to C–7, in combination with the COSY (COrrelated SpectroscopY) correlation between 5/9–H and 6/8–H, revealed the presence of an *O*-methyl-2,3-dehydrotyrosine moiety. A ROESY (ROtating-frame NOE SpectroscopY) correlation between 2–NH and 5/9–H indicated the Δ^2,3^ double bond to be in *Z*-configuration. All other signals belong to a monounsaturated fatty acid moiety, which is connected by an amide linkage due to the HMBC correlation from 2–NH to C1′. The ^13^C-NMR chemical shifts δ_c_ 26.5 of the allylic methylenes C–8′/C–11′ are characteristic for a *cis*-double bond geometry (Alexandri et al., 2017). Since it was impossible to locate the double bond by NMR data, a fatty acid methyl ester analysis by GC-MS was performed. Comparison with authentic standards revealed the C_18_ fatty acid to be oleic acid (*cis-Δ*^9^-octadecenoic acid).

**TABLE 1:** NMR Spectroscopic Data (^1^H 700 MHz, ^13^C 175 MHz, DMSO-*d*_6_) for **1**.

Pos.	δ _C_, Type	δ _H_ (*J* in Hz)	COSY	HMBC	ROESY
1	166.6, C				
2	125.1, C				
3	131.5, CH	7.21, s		1	
4	126.3, C				
7	159.9, C				
6/8	113.9, CH	6.93, d (8.8)	5/9	4,6/8,7	5/9,10
5/9	131.5, CH	7.57, d (8.8)	6/8	3,5/9,7	3, 6/8
10	55.2, CH_3_	3.77, s		7	6/8
1’	171.9, C				
2’	33.6, CH_2_	2.18, t (7.4)	3′	1′,3′,4′	2NH, 3′
3’	24.5, CH_2_	1.47, m	2′,4′	1′,2′,4′	2′,4′
4′-7′	28.5–29.0, CH_2_	1.26, m^a^			
9’	129.63, CH	5.32, t (4.6)^b^	8′		
10’	129.60, CH	5.32, t (4.6)^b^	11′		
11′, 8′	26.5, CH_2_	1.98, m			
12′-15′	28.5–29.0, CH_2_	1.26, m^a^			
16’	31.2, CH_2_	1.26, m^a^			
17’	22.1, CH_2_	1.26, m^a^			
18’	13.9,CH_3_	0.85, t (7.1)	17′	16′,17′	17′
NH		9.26, s			

*^a,b^Overlapping signals.*

The structure of **1** (Figure 3) largely resembles thalassotalic acids from the marine γ-proteobacterium *Thalassotalea* sp. PP2-459 (Deering et al., 2016) and stieleriacines A-C from *S. maiorica* Mal15^T^ (Kallscheuer et al., 2020). Differences were observed in the length of the fatty acid chain and the methylation pattern of the aromatic ring in the tyrosine moiety. In accordance with the assigned genus of strain Enr13^T^, **1** was named stieleriacine D.

**FIGURE 4:**
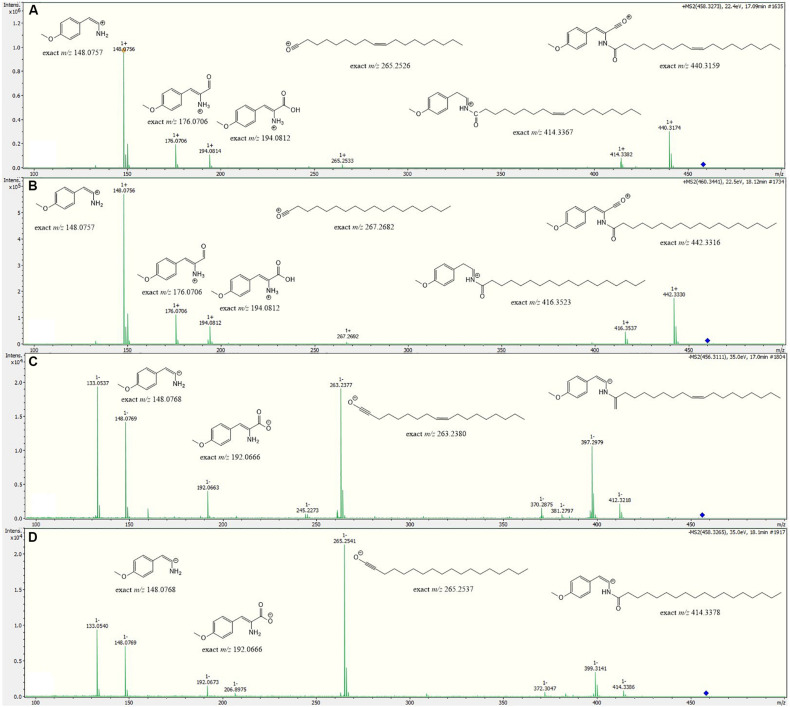
HRESIMS/MS spectra of stieleriacine D (**1**) and E (**2**) in positive and negative modes with potential fragments. **(A) 1** in positive mode **(B) 2** in positive mode **(C) 1** in negative mode **(D) 2** in negative mode.

Metabolite **2** was detected in the chromatogram of the crude extract of strain Enr13^T^ in proximity to stieleriacine D (**1**). HRESIMS data showed a molecular ion cluster of [M+H]^+^ *m/z* 460.3418, corresponding to a molecular formula of C_28_H_45_NO_4_, suggesting two additional hydrogen atoms compared to **1**. Isolation attempts did not yield a sufficient amount of the compound in question and therefore did not allow for a structure elucidation by NMR spectroscopy. However, the metabolite could be characterized via MS/MS analysis (Figure 4). Fragments were observed for the cleavage of the amide bond. An MS^2^ peak of *m/z* 194.0811 [M+H]^+^ in the positive mode indicated the presence of an *O*-methyl-dehydrotyrosine fragment for both **1** and **2**, demonstrating this moiety to be unaltered in **2** compared to **1**. However, peaks at *m/z* 265.2534 [M-H]^+^ corresponding to an oleyl fragment for **1** and at *m/z* 267.2692 [M-H]^+^ corresponding to a stearyl fragment for **2**, identified the fatty acid moiety of **2** as stearic acid (octadecanoic acid). MS^2^ analysis in the negative mode supported the assignment, with the same amino acid fragment observed at *m/z* 192.0663 for both **1** and **2**, but fragments at *m/z* 263.2377 and 265.2537 for fatty acids. Accordingly, we named this metabolite stieleriacine E (**2**).

#### Biological Activity

The bioactivity of stieleriacine D (**1**) was assessed with our standard panel of test organisms (see Supplementary Table S3). It exhibited antibiotic activity against the Gram-positive bacteria *Micrococcus luteus* (MIC = 16.7 μg/mL) and *Staphylococcus aureus* (MIC = 66.7 μg/mL). It did not display activity against Gram-negative bacteria and fungi, nor any cytotoxicity against tested cell lines L929 and KB3.1 up to 10 μM (data not shown).

#### A Putative Biosynthetic Route to Stieleriacine D

Long-chain *N*-acyl amino acids such as stieleriacine D identified in this study are likely produced from the respective long-chain fatty acid and an amino acid derivative. The fatty acid serves as a substrate in form of its acyl carrier protein (ACP)-activated thioester (acyl-ACP). The amino acid residue can be a canonical or non-canonical amino acid, which can be further modified (“decorated”). Dedicated *N*-acyl amino acid synthases (NASs) catalyze the ligation of the fatty acid residue to the amino group of the amino acid derivative yielding an amide group in the ligation product (Brady et al., 2002). In case of stieleriacine D, the two compounds likely serving as precursors are oleoyl-ACP (the ACP-thioester of oleic acid) and *O*-methyl-tyrosine. Oleic acid is a naturally occuring fatty acid and is probably directly consumed from the primary metabolism of strain Enr13^T^. Accordingly, the amino acid substrate is obtained by *O*-methylation of the aromatic amino acid L-tyrosine.

**FIGURE 5:**
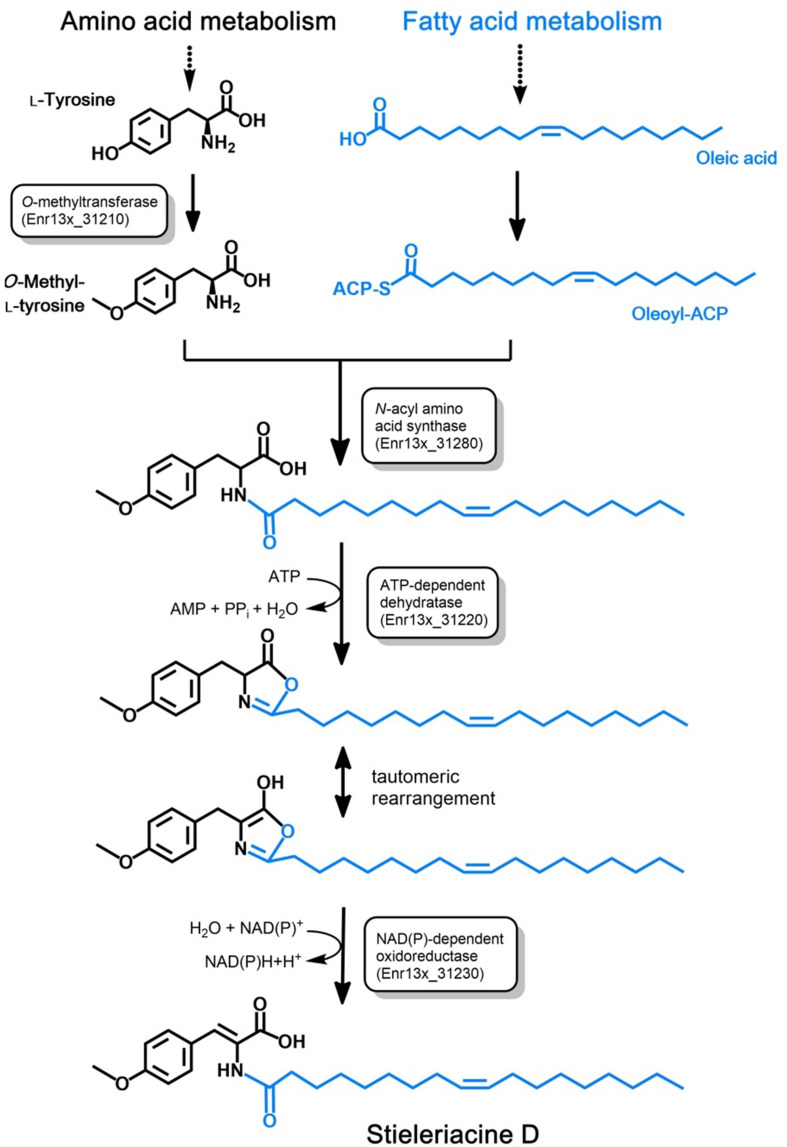
Postulated pathway for stieleriacine D biosynthesis. The proposed stieleriacine D biosynthesis pathway starting from L-tyrosine and oleic acid in strain Enr13^T^ is depicted. Enzymatic activities for the required reactions steps are shown in boxes. The Enr13^T^ gene locus tag is given for putative enzymes with the expected activities that were found in the postulated gene cluster. ACP: acyl carrier protein.

**FIGURE 6:**
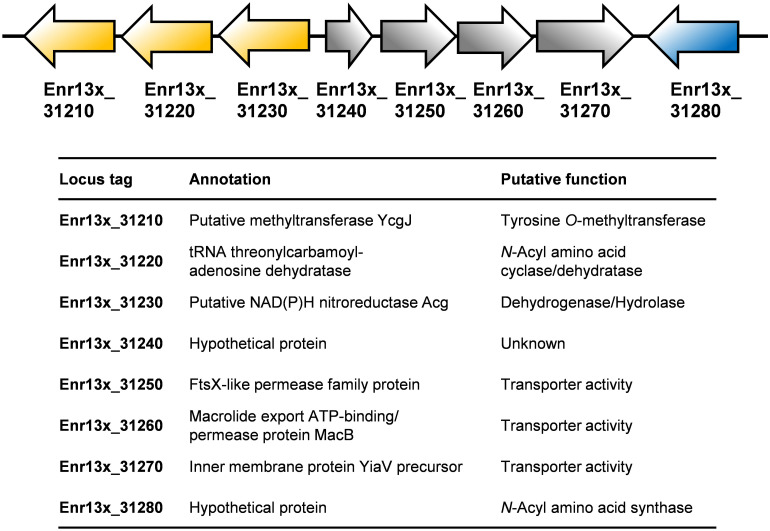
Putative stieleriacine biosynthetic gene cluster. The organization of the genes in the cluster is depicted. For encoded enzymes, the putative enzyme function/protein activity is given. Same colors do not indicate polycistronic operons (no information on the expression pattern is available).

Based on an *in silico* constructed putative biosynthetic route for stieleriacine D (Figure 5), we searched for clusters in the genome of strain Enr13^T^ potentially encoding enzymes of this pathway (Figure 6). The analysis was performed manually using Blastp and InterproScan based on known sequences of the key enzyme NAS and was additionally guided by automated prediction of biosynthesis gene clusters by AntiSMASH (in which *N*-acyl amino acid prediction was recently implemented). The analysis yielded three putative NASs (NasY homologs) encoded in the genome of strain Enr13^T^ (locus tags Enr13x_30590, Enr13x_31280, and Enr13x_41680), which were all annotated as “hypothetical proteins” by the automated annotation after genome sequencing. One of the putative NAS-encoding genes (Enr13x_31280) was found to be encoded seven genes downstream of Enr13x_31210, which was identified as the best gene candidate for the tyrosine *O*-methyltransferase (*S*-adenosyl methionine-dependent methyltransferase class I;YcgJ homolog). This finding supported the notion that enzymes required for biosynthesis of stieleriacine D are encoded in this cluster.

Stieleriacines can harbor a double bond in 2,3-position of the tyrosine moiety, which is also present in thalassotalic acids identified in *Thalassotalea* sp. PP2-459 (Deering et al., 2016). For both classes of compounds, it has not yet been elucidated how and at which stage of the biosynthetic pathway the double bond is actually introduced. Enr13x_31220, encoded immediately downstream of the methyltransferase, codes for a putative tRNA threonylcarbamoyladenosine dehydratase. The dehydratase naturally catalyzes the ATP-dependent lactonization and subsequent dehydration of threonylcarbamoyladenosine. This substrate resembles the amide group present in stieleriacines. The protein in strain Enr13^T^ might thus catalyze a similar reaction in the stieleriacine pathway (Figure 5). Subsequent oxidation and hydrolysis lead to the production of stieleriacine D, which could be catalyzed by the putative oxidoreductase encoded by Enr13x_31230 located downstream of the dehydratase gene. Other genes in the cluster (Enr13x_31240-70) code for putative permease and exporter proteins, which might be relevant for secretion of stieleriacines, however, we cannot provide any additional evidence at this early stage of research. Enr13x_31240 codes for a putative protein of only 88 amino acids and may thus be erroneously annotated.

#### Comparison to Known Secondary Metabolites

Stieleriacines D (**1**) and E (**2**) contain a 2,3-dehydrotyrosine moiety as a characteristic feature. 2,3-dehydroamino acids are naturally occurring non-coding amino acids, which were found as part of various peptides (Siodłak, 2015). These peptides were isolated primarily from bacteria and less often from fungi, marine invertebrates or higher plants. The compounds showed antibiotic, antifungal, antitumor and/or phytotoxic activity.

Besides the thalassotalic acids from the marine bacterium *Thalassotalea* sp. PP2-459 (Deering et al., 2016), stieleriacines have a striking similarity to *N*-acylated derivatives of amino acids, including tyrosine, which have previously been found by heterologous expression of environmental DNA in *Escherichia coli* (Brady et al., 2002; Brady and Clardy, 2005). NAS-derived products have even been found with commensal human bacteria. Their influence on G-protein-coupled receptors suggested a chemical mimicry of eukaryotic metabolites (Cohen et al., 2017).

Together with the high frequency of detection in environmental DNA libraries, this fact indicates an important role of *N*-acylated amino acid derivatives in signaling processes in general. Over the last few years, a number of compounds similar to stieleriacines were identified (Deering et al., 2016; Lee et al., 2019; MacIntyre et al., 2019). Previously published data together with the characterization of stieleriacines in this study will be the basis for further elucidating the underlying biosynthetic routes including their regulation as well as the natural role of these bioactive small molecules. Since the characterization of these metabolites is often hampered by low production titers, the knowledge of MS/MS fragmentation will simplify the detection and assignment of secondary metabolites of this substance family, by facilitating MS/MS networking-guided analyses (Nguyen et al., 2013).

## Materials and Methods

### Sample Collection and Preparation

Strain Enr13^T^ was isolated from the biofilm of a *Posidonia* sp. leaf collected close to the island Panarea, Italy (exact location 38.6457 N 15.0772 E). Sampling took place on the 9th of September 2013. A *Posidonia* leaf was cut off from the plant, placed in a Falcon tube and stored at 4°C until further processing. In the laboratory, the leaf was washed with 0.5x artificial seawater (ASW). ASW (1x) contained 46.94 g/L NaCl, 7.84 g/L Na_2_SO_4_, 21.28 g/L MgCl_2_ ⋅ 6 H_2_O, 2.86 g/L CaCl_2_ ⋅ 2 H_2_O, 0.384 g/L NaHCO_3_, 1.384 g/L KCl, 0.192 g/L KBr, 0.052 g/L H_3_BO_3_, 0.08 g/L SrCl_2_ ⋅ 6 H_2_O, and 0.006 g/L NaF. Subsequently, the biofilm was scraped off from the leaf surface using a single-use scalpel and the biofilm was resuspended in 20 μL 0.5x ASW. The suspension was thoroughly mixed and streaked on a plate containing M1H NAG ASW medium solidified with 8 g/L gellan gum (Wiegand et al., 2020), additionally supplemented with 500 mg/L streptomycin, 200 mg/L carbenicillin and 20 mg/L cycloheximide. The plate was cultivated at 20°C for 6 weeks and was regularly inspected for growth of bacterial colonies.

### Culture and Isolation Conditions

M1 medium with HEPES (H) as buffering agent and additionally supplemented with *N*-acetyl glucosamine (NAG) and ASW was used (medium designated as M1H NAG ASW) (Wiegand et al., 2020). It contained 250 mL/L ASW (46.94 g/L NaCl, 7.84 g/L Na_2_SO_4_, 21.28 g/L MgCl_2_ ⋅ 6 H_2_O, 2.86 g/L CaCl_2_ ⋅ 2 H_2_O, 0.384 g/L NaHCO_3_, 1.384 g/L KCl, 0.192 g/L KBr, 0.052 g/L H_3_BO_3_, 0.08 g/L SrCl_2_ ⋅ 6 H_2_O, and 0.006 g/L NaF), supplemented with 0.25 g/L peptone (Bacto), 0.25 g/L yeast extract (Bacto), 20 mL/L mineral salts solution, 1 g/L *N*-acetyl glucosamine, 0.25 g/L glucose and 5 mL/L vitamin solution. In addition, 1 mL trace element solution (1.5 g/L Na–nitrilotriacetate, 500 mg/L MnSO_4_ ⋅ H_2_O, 100 mg/L FeSO_4_ ⋅ 7 H_2_O, 100 mg/L Co(NO_3_)_2_ ⋅ 6 H_2_O, 100 mg/L ZnCl_2_, 50 mg/L NiCl_2_ ⋅ 6 H_2_O, 50 mg/L H_2_SeO_3_, 10 mg/L CuSO_4_ ⋅ 5 H_2_O, 10 mg/L AlK(SO_4_)_2_ ⋅ 12 H_2_O, 10 mg/L H_3_BO_3_, 10 mg/L NaMoO_4_ ⋅ 2 H_2_O and 10 mg/L Na_2_WO_4_ ⋅ 2 H_2_O) as well as 1 mL/L – of each 100 mg/mL carbenicillin and 20 mg/mL cycloheximide – stock solutions were added. The medium was buffered at pH 8.0 with 10 mM HEPES. Solid media were prepared with Bacto agar (12 g/L, washed three times with ddH_2_O), cooled to 55°C after autoclaving before adding heat-sensitive solutions. The mineral salt solution as well as vitamin solution were prepared according to DSMZ medium 621, while metal salts solution consisted of 250 mg/L Na_2_–EDTA, 1095 mg/L ZnSO_4_ ⋅ 7 H_2_O, 500 mg/L FeSO_4_ ⋅ 7 H_2_O, 154 mg/L MnSO_4_ ⋅ H_2_O, 39.5 mg/L CuSO_4_ ⋅ 5 H_2_O, 20.3 mg/L CoCl_2_ ⋅ 6 H_2_O, and 17.7 mg/L Na_2_B_4_O_7_⋅10 H_2_O of which 50 mL were added per liter of mineral salt solution. Pure cultures were cryopreserved in M1H NAG ASW medium with 50% glycerol or 5% DMSO and stored at −80°C.

### Morphological Analysis

Enr13^T^ cells were immobilized on a 1% agarose–pad in MatTek Glass Bottom Microwell Dishes (35 mm dish, 14 mm microwell with No. 1.5 cover-glass P35G-1.5-14-C) and were imaged with phase–contrast (Phaco) illumination using a NikonTi microscope at 100x magnification with a Nikon N Plan Apochromat λ 100x/1.45 oil objective and the Nikon DS–Ri2 camera (blue LED). To determine the cell size of the novel strain, 500 individual cells were measured using the object count tool of NIS-Elements software V4.3 (Nikon Instruments).

Scanning electron microscopy (SEM) was performed as previously described (Rast et al., 2017). Briefly, samples were placed onto poly-L-lysine-coated cover slips (12 mm) for 10 min, then fixed with 2% glutaraldehyde in TE buffer (10 mM TRIS, 1 mM EDTA, pH 6.9) and dehydrated with a graded series of acetone (10, 30, 50, 70, 90, 100%) on ice 10 min for each step. After critical point drying with CO_2_, samples were mounted onto aluminum stubs with adhesive tape, sputter coated with gold-palladium and examined in a Zeiss Merlin field emission scanning electron microscope. Images were taken with the SEM software version 5.05 at an acceleration voltage of 5 kV with the Inlens SE-detector and HESE2 SE-detector in a 75:25 ratio.

### Physiological Tests

Physiological tests such as cultivations for determination of pH and temperature tolerance were performed in M1H NAG ASW. Cells were inoculated 1:10 from early stationary phase cultures in glass tubes and incubated under constant agitation. To determine the pH optimum, M1H NAG ASW medium was adjusted to pH values of 5.0, 6.0, 6.5, 7.0, 7.5, 8.0, 8.5, 9.0, and 10.0 (Supplementary Figure S1) using 100 mM MES, HEPES, HEPPS and CHES buffers, corresponding to their individual buffer range. Cultivations were done at a constant temperature of 28°C (variation of pH) or at pH 8.0 (variation of temperature). Measurements were performed in triplicates and each tube served as its own blank prior to inoculation. Growth was detected by monitoring the optical density at 600 nm (OD_600_) using a Photometer Ultrospec II (LKB Biochrom). Growth rates (in h^–1^) were obtained as the slope from the linear range of a plot of ln(OD_600_) against the cultivation time.

Catalase activity was determined by bubble formation with fresh 3% H_2_O_2_ solution. Cytochrome oxidase activity was determined using Bactident Oxidase test stripes (Merck Millipore) following the manufacturer’s instructions.

### Phylogenetic Analysis, Genome Information and Genome-Based Analysis

Novel isolates were identified by direct sequencing of the 16S rRNA gene after amplification with the optimized universal primers 8f (5′ AGA GTT TGA TCM TGG CTC AG–3′) and 1492r (5′–GGY TAC CTT GTT ACG ACT T–3′) modified from Wiegand et al. (2020). PCR reactions were performed directly on single colonies for identification or on liquid cultures to check for purity, using the Taq DNA Polymerase Kit (Qiagen) with one reaction of 25 μL containing 11 μL PCR–grade H_2_O, 2.5 μL 10x CoralLoad buffer, 2.5 μL Q-Solution, 0.5 μL dNTPs (10 mM each), 1 μL sterile bovine serum albumin solution (20 mg/mL), 0.5 μL MgCl_2_ solution (25 mM), 0.125 μL Taq–Polymerase (1 U/μL) and 1 μL of each primer (10 pmol). The employed protocol consisted of two steps, the first step with an initial denaturation at 94°C, 5 min, 10 cycles of denaturation at 94°C, 30 s, annealing at 59°C, 30 s, elongation at 72°C, 1 min, followed by the second step with 20 cycles denaturation at 94°C, 30 s, annealing at 54°C, 30 s, elongation at 72°C, 1 min and a final elongation step at 72°C, 7 min. All PCRs were carried out in an Applied Biosystems Veriti thermal cycler (Thermo Fisher Scientific) and PCR products were stored at 4°C until Sanger sequencing.

The genome of strain Enr13^T^ was previously published (Wiegand et al., 2020) and is available from GenBank under accession no. CP037423. The accession no. of the 16S rRNA gene used for the phylogenetic analysis is MK559971. 16S rRNA gene sequence- and MLSA-based phylogeny was computed for strain Enr13^T^, the type strains of all described planctomycetal species (as in January 2020) and all isolates recently published (Wiegand et al., 2020). Proteins putatively involved in the stieleriacine biosynthesis pathway were analyzed using InterProScan (Jones et al., 2014). Blastp analysis was performed using proteins with known function. Blastp and InterproScan were used with ‘default parameters’. AntiSMASH analysis (bacterial version 5.1.2) was performed based on GenBank accession no. CP037423 with relaxed strictness and the following extra features enabled: KnownClusterBlast, ActiveSiteFinder and SubClusterBlast (Blin et al., 2019).

### Fermentation and Isolation of Metabolites

For the isolation of secondary metabolites from strain Enr13^T^, the slightly nutrient-richer medium M3H NAG ASW was used, containing an extra 1.0 g/L peptone (Bacto), and 1.0 g/L yeast extract (Bacto). Enr13^T^ cultures were incubated in six 2 L baffled flasks, each containing 800 mL culture solution, and cultivated at 28°C and 80 rpm. After 1 day, 2% (v/v) purified adsorbent resin XAD-16N (Rohm and Haas) was added and the cultures were incubated for another 7 days. Afterward, the XAD was harvested through separation and extracted with acetone (Jeske et al., 2013). The obtained crude extract was evaporated to dryness (36°C), re-suspended in methanol/water (1:1) and filtered through a Strata-X 33 mm, Polymeric Reverse Phase Solid-Phase cartridge (Phenomenex, Aschaffenburg, Germany). The filtrate was disposed and the solid-phase eluted with acetone and hexane yielding 129.5 mg of crude product. The crude product was fractionated using preparative RP-HPLC (Gilson, Middleton, WI, United States). A Luna C_18_(2) column (250 × 21 mm, 7 μm [Phenomenex, Aschaffenburg, Germany]) served as stationary phase. Deionized water (MilliQ, Darmstadt, Germany) with 0.05% trifluoroacetic acid (TFA; solvent A), and acetonitrile with 0.05% TFA (solvent B), were used as mobile phases. The elution gradient started at 10% B for 10 min, increasing to 100% B within 50 min; followed by 10 min at 100% B. UV detection was carried out at 220 and 300 nm. Fractions were collected and combined yielding 1.0 mg of stieleriacine D (**1**).

*Stieleriacine D* (**1**): off-white, amorphous solid; UV (MeOH) λ_max_ (log ε) 202 (4.0), 298 (3.9); ^1^H and ^13^C NMR data in DMSO-*d*_6_ see Table 1; HRESIMS 458.3282 [M+H]^+^ (calcd. for C_28_H_44_${\text{NO}}_{4}^{+}$, 458.3270).

### Structure Elucidation

HRESIMS mass spectra were measured with an Agilent 1200 series HPLC-UV system in combination with an ESI-TOF-MS (Maxis, Bruker) [column 2.1 × 50 mm, 1.7 μm, C18 Acquity UPLC BEH (Waters), solvent A: H_2_O + 0.1% formic acid, solvent B: acetonitrile + 0.1% formic acid, gradient: 5% B for 0.5 min increasing to 100% B in 19.5 min, maintaining 100% B for another 5 min, RF = 0.6 mL min^–1^, UV detection 200 – 600 nm]. NMR spectra were recorded on a Bruker Avance III 500 MHz spectrometer with a BBFO(plus) SmartProbe (^1^H 500 MHz, ^13^C 126 MHz), and a Bruker Avance III 700 MHz spectrometer with a 5 mm TCI cryoprobe (^1^H 700 MHz, ^13^C 175 MHz, ^15^N 71 MHz). Chemical shifts *δ* were referenced to DMSO-*d*_6_ (^1^H, *δ* = 2.50 ppm; ^13^C, *δ* = 39.51 ppm). UV spectra were recorded using the Shimadzu UV*vis* spectrophotometer UV-2450.

### Fatty Acid Methyl Ester Analysis

Stieleriacine D (0.5 mg) was saponified using 0.9 mL methanolic NaOH (MeOH : NaOH 15%, 1:1) at 100°C for 1 h. Afterward, 1.8 mL methanolic HCl (MeOH : HCl 37%; 10:2) was added at room temperature and heated for 10 min at 80°C, followed by immediate cooling on ice. 0.9 mL hexane/methyl-*tert*-butyl ether (1:1; v:v) were added and mixed for 30 s. The organic layer was then transferred into a new vial and the extraction step repeated twice. 2.5 mL of a 0.5 M NaOH solution were subsequently added to the organic phase and mixed for 30 s. The organic layer was then transferred into a GC vial, evaporated and re-suspended in 500 μL octane and measured using the Agilent 6890N GC with FID (flame ionization detector). Separation of the fatty acid methyl esters was achieved with an Optima 5 column (5% phenyl, 95% dimethylpolysiloxane; 50 m length; 0.32 mm inner diameter; 0.25 μm film thickness; Macherey-Nagel, Düren, Germany). Individual fatty acid methyl esters were identified by comparison of their retention time with standards.

### Minimum Inhibitory Concentrations

Minimum Inhibitory Concentrations (MIC) were investigated in a serial dilution assay in 96-well microtiter plates in YM medium for yeasts and filamentous fungi and BD^TM^ Difco^TM^ Müller-Hinton Broth for bacteria, as previously published (Surup et al., 2015).

### Cytotoxicity Assay

The *in vitro* cytotoxicity assay was carried out against the mouse fibroblasts cell line L929 and HeLa (KB3.1) cells as described earlier (Sandargo et al., 2019).

## Conclusion

The strain Enr13^T^ was characterized as *Stieleria neptunia* sp. nov., a new species of the *Planctomycetes* phylum. From extracts of this strain we isolated two new secondary metabolites, which we characterized by NMR and HRESIMS(/MS). By analyzing the potential biosynthetic gene cluster responsible for their production, we proposed a biosynthetic pathway.

## Data Availability Statement

This genome of strain Enr13^T^ was published previously (Wiegand et al., 2020) and is available from GenBank under accession no. CP037423. The accession no. of the 16S rRNA gene used for the phylogenetic analysis is MK559971. AntiSMASH analysis (bacterial version 5.1.2) was performed based on GenBank accession no. CP037423.

## Author Contributions

BS, OJ, NK, and FS prepared the original draft. OJ carried out the visualization and validation of biological experiments. BS contributed to most chemical experiments. BS and FS contributed to structure elucidation. BS and J-PW contributed to MS/MS spectrometry. CB contributed to time-lapse- and general light microscopy. SW contributed to phylogeny. NK contributed to pathway analysis. CJ and MJ contributed to strain isolation. MR contributed to the electron micrographs. FS and CJ contributed to supervision, project administration, and funding acquisition. All authors reviewed and edited the manuscript.

## Conflict of Interest

The authors declare that the research was conducted in the absence of any commercial or financial relationships that could be construed as a potential conflict of interest.

## References

[B1] Alexandri E. Ahmed R. Siddiqui H. Choudhary M. I. Tsiafoulis C. G. Gerothanassis I. P. (2017). High resolution NMR spectroscopy as a structural and analytical tool for unsaturated lipids in solution. *Molecules* 22: E1663. 10.3390/molecules22101663 PMC6151582 28981459

[B2] Blin K. Shaw S. Steinke K. Villebro R. Ziemert N. Lee S. Y. (2019). antiSMASH 5.0: updates to the secondary metabolite genome mining pipeline. *Nucleic Acids Res.* 47 W81–W87. 31032519 10.1093/nar/gkz310 PMC6602434

[B3] Boedeker C. Schuler M. Reintjes G. Jeske O. van Teeseling M. C. Jogler M. (2017). Determining the bacterial cell biology of Planctomycetes. *Nat. Commun.* 8:14853. 10.1038/ncomms14853 PMC5394234 28393831

[B4] Bondoso J. Harder J. Lage O. M. (2013). *rpoB* gene as a novel molecular marker to infer phylogeny in *Planctomycetales*. *Antonie Van Leeuwenhoek* 104 477–488. 10.1007/s10482-013-9980-7 23904187

[B5] Brady S. F. Chao C. J. Clardy E. (2002). New natural products families from an environmental DNA (eDNA) gene cluster. *J. Am. Chem. Soc.* 124 9968–9969. 10.1021/ja0268985 12188643

[B6] Brady S. F. Clardy J. (2005). N-Acyl derivatives of arginine and tryptophan isolated from environmental DNA expressed in *Escherichia coli*. *Org. Lett.* 7 3613–3616. 10.1021/ol0509585 16092832

[B7] Calisto R. Sæbø E. F. Storesund J. E. Øvreås L. Herfindal L. Lage O. M. (2019). Anticancer activity in Planctomycetes. *Front. Mar. Sci.* 5:499 10.3389/fmars.2018.00499

[B8] Cohen L. J. Esterhazy D. Kim S. H. Lemetre C. Aguilar R. R. Gordon E. A. (2017). Commensal bacteria make GPCR ligands that mimic human signalling molecules. *Nature* 549 48–53. 10.1038/nature23874 28854168 PMC5777231

[B9] Deering R. W. Chen J. Sun J. Ma H. Dubert J. Barja J. L. (2016). N-acyl dehydrotyrosines, tyrosinase inhibitors from the marine bacterium *Thalassotalea* sp. *PP*2-459. *J. Nat. Prod.* 79 447–450. 10.1021/acs.jnatprod.5b00972 26824128 PMC5821419

[B10] Delmont T. O. Quince C. Shaiber A. Esen ÖC. Lee S. T. Rappé M. S. (2018). Nitrogen-fixing populations of *Planctomycetes* and *Proteobacteria* are abundant in surface ocean metagenomes. *Nat. Microbiol.* 3 804–813. 10.1038/s41564-018-0176-9 29891866 PMC6792437

[B11] Devos D. P. (2014). Re-interpretation of the evidence for the PVC cell plan supports a Gram-negative origin. *Antonie Van Leeuwenhoek* 105 271–274. 10.1007/s10482-013-0087-y 24292377

[B12] Faria M. Bordin N. Kizina J. Harder J. Devos D. Lage O. M. (2018). Planctomycetes attached to algal surfaces: insight into their genomes. *Genomics* 110 231–238. 10.1016/j.ygeno.2017.10.007 29074368

[B13] Frank O. Michael V. Pauker O. Boedeker C. Jogler C. Rohde M. (2014). Plasmid curing and the loss of grip – The 65-kb replicon of *Phaeobacter inhibens* DSM 17395 is required for biofilm formation, motility and the colonization of marine algae. *Syst. Appl. Microbiol.* 38 120–127. 10.1016/j.syapm.2014.12.001 25595869

[B14] Godinho O. Calisto R. Øvreås L. Quinteira S. Lage O. M. (2019). Antibiotic susceptibility of marine Planctomycetes. *Antonie Van Leeuwenhoek* 112 1273–1280. 10.1007/s10482-019-01259-7 30919144

[B15] Graça A. P. Calisto R. Lage O. M. (2016). Planctomycetes as novel source of bioactive molecules. *Front. Microbiol.* 7:1241. 10.3389/fmicb.2016.01241 27570520 PMC4982196

[B16] Jeske O. Jogler M. Petersen J. Sikorski J. Jogler C. (2013). From genome mining to phenotypic microarrays: planctomycetes as source for novel bioactive molecules. *Antonie Van Leeuwenhoek* 104 551–567. 10.1007/s10482-013-0007-1 23982431

[B17] Jeske O. Schüler M. Schumann P. Schneider A. Boedeker C. Jogler M. (2015). Planctomycetes do possess a peptidoglycan cell wall. *Nat. Commun.* 6:7116. 10.1038/ncomms8116 PMC4432640 25964217

[B18] Jeske O. Surup F. Ketteniß M. Rast P. Förster B. Jogler M. (2016). Developing techniques for the utilization of planctomycetes as producers of bioactive molecules. *Front. Microbiol.* 7:1242. 10.3389/fmicb.2016.01242 27594849 PMC4990742

[B19] Jogler C. Glöckner F. O. Kolter R. (2011). Characterization of *Planctomyces limnophilus* and development of genetic tools for its manipulation establish it as a model species for the phylum *Planctomycetes*. *Appl. Environm. Microbiol.* 77 5826–5829. 10.1128/aem.05132-11 21724885 PMC3165242

[B20] Jogler C. Waldmann J. Huang X. Jogler M. Glöckner F. O. Mascher T. (2012). Identification of proteins likely to be involved in morphogenesis, cell division, and signal transduction in Planctomycetes by comparative genomics. *J. Bacteriol.* 194 6419–6430. 10.1128/jb.01325-12 23002222 PMC3497475

[B21] Jones P. Binns D. Chang H. Y. Fraser M. Li W. McAnulla C. (2014). InterProScan 5: genome-scale protein function classification. *Bioinformatics* 30 1236–1240. 10.1093/bioinformatics/btu031 24451626 PMC3998142

[B22] Kallscheuer N. Jeske O. Sandargo B. Boedeker C. Wiegand S. Bartling P. (2020). The planctomycete *Stieleria maiorica* Mal15^T^ employs stieleriacines to alter the species composition in marine biofilms. *Commun. Biol.* 3:303. 10.1038/s42003-020-0993-2 32533057 PMC7293339

[B23] Kim M. Oh H.-S. Park S.-C. Chun J. (2014). Towards a taxonomic coherence between average nucleotide identity and 16S rRNA gene sequence similarity for species demarcation of prokaryotes. *Int. J. Syst. Evolut. Microbiol.* 64 346–351. 10.1099/ijs.0.059774-0 24505072

[B24] Kohn T. Wiegand S. Boedeker C. Rast P. Heuer A. Jetten M. S. M. (2020). *Planctopirus ephydatiae*, a novel Planctomycete isolated from a freshwater sponge. *Syst. Appl. Microbiol.* 43:126022. 10.1016/j.syapm.2019.126022 31785948

[B25] Konstantinidis K. T. Tiedje J. M. (2005). Genomic insights that advance the species definition for prokaryotes. *Proc. Natl. Acad. Sci. U.S.A.* 102 2567–2572. 10.1073/pnas.0409727102 15701695 PMC549018

[B26] Lage O. M. Bondoso J. (2014). Planctomycetes and macroalgae, a striking association. *Front. Microbiol.* 5:267. 10.3389/fmicb.2014.00267 24917860 PMC4042473

[B27] Lee C.-M. Kim S.-Y. Yoon S.-H. Kim J.-B. Yeo Y.-S. Sim J.-S. (2019). Characterization of a novel antibacterial N-acyl amino acid synthase from soil metagenome. *J. Biotechnol.* 294 19–25. 10.1016/j.jbiotec.2019.01.017 30771442

[B28] MacIntyre L. W. Charles M. J. Haltli B. A. Marchbank D. H. Kerr R. G. (2019). An Ichip-domesticated sponge bacterium produces an N-acyltyrosine bearing an α-methyl substituent. *Org. Lett.* 21 7768–7771. 10.1021/acs.orglett.9b02710 31524403

[B29] Nguyen D. D. Wu C. H. Moree W. J. Lamsa A. Medema M. H. Zhao X. (2013). MS/MS networking guided analysis of molecule and gene cluster families. *Proc. Natl. Acad. Sci. U.S.A.* 110 E2611–E2620. 23798442 10.1073/pnas.1303471110 PMC3710860

[B30] Overmann J. Abt B. Sikorski J. (2017). Present and future of culturing bacteria. *Annu. Rev. Microbiol.* 71 711–730. 10.1146/annurev-micro-090816-093449 28731846

[B31] Panter F. Garcia R. Thewes A. Zaburannyi N. Bunk B. Overmann J. (2019). Production of a dibrominated aromatic secondary metabolite by a planctomycete implies complex interaction with a macroalgal host. *ACS Chem. Biol.* 14 2713–2719. 10.1021/acschembio.9b00641 31644258

[B32] Qin Q.-L. Xie B.-B. Zhang X.-Y. Chen X.-L. Zhou B.-C. Zhou J. (2014). A proposed genus boundary for the prokaryotes based on genomic insights. *J. Bacteriol.* 196 2210–2215. 10.1128/jb.01688-14 24706738 PMC4054180

[B33] Rast P. Glockner I. Boedeker C. Jeske O. Wiegand S. Reinhardt R. (2017). Three novel species with peptidoglycan cell walls form the new genus *Lacunisphaera* gen. nov. in the family Opitutaceae of the verrucomicrobial subdivision 4. *Front. Microbiol.* 8:202. 10.3389/fmicb.2017.00202 28243229 PMC5303756

[B34] Ravin N. V. Rakitin A. L. Ivanova A. A. Beletsky A. V. Kulichevskaya I. S. Mardanov A. V. (2018). Genome analysis of *Fimbriiglobus ruber* SP5^T^, a planctomycete with confirmed chitinolytic capability. *Appl. Environ. Microbiol.* 84:e02645-17. 10.1128/AEM.02645-17 29374042 PMC5861812

[B35] Rivas-Marin E. Canosa I. Santero E. Devos D. P. (2016). Development of genetic tools for the manipulation of the Planctomycetes. *Front. Microbiol.* 7:914. 10.3389/fmicb.2016.00914 27379046 PMC4910669

[B36] Sandargo B. Michehl M. Praditya D. Steinmann E. Stadler M. Surup F. (2019). Antiviral meroterpenoid rhodatin and sesquiterpenoids rhodocoranes A–E from the wrinkled peach mushroom, *Rhodotus palmatus*. *Org. Lett.* 21 3286–3289. 10.1021/acs.orglett.9b01017 31008606

[B37] Siodłak D. (2015). α,β-Dehydroamino acids in naturally occurring peptides. *Amino Acids* 47 1–17. 10.1007/s00726-014-1846-4 25323736 PMC4282715

[B38] Stackebrandt E. Ebers J. (2006). Taxonomic parameter revisited: tarnished gold standards. *Microbiol. Today* 33 152–155.

[B39] Surup F. Thongbai B. Kuhnert E. Sudarman E. Hyde K. D. Stadler M. (2015). Deconins A-E: cuparenic and mevalonic or propionic acid conjugates from the basidiomycete *Deconica* sp. 471. *J. Nat. Prod.* 78 934–938. 10.1021/np5010104 25871540

[B40] van Teeseling M. C. Mesman R. J. Kuru E. Espaillat A. Cava F. Brun Y. V. (2015). Anammox Planctomycetes have a peptidoglycan cell wall. *Nat. Commun.* 6:6878. 10.1038/ncomms7878 PMC4432595 25962786

[B41] Wiegand S. Jogler M. Boedeker C. Pinto D. Vollmers J. Rivas-Marín E. (2020). Cultivation and functional characterization of 79 planctomycetes uncovers their unique biology. *Nat. Microbiol.* 5 126–140. 10.1038/s41564-019-0588-1 31740763 PMC7286433

[B42] Wiegand S. Jogler M. Jogler C. (2018). On the maverick Planctomycetes. *FEMS Microbiol. Rev.* 42 739–760. 10.1093/femsre/fuy029 30052954

[B43] Yadav S. Vaddavalli R. Siripuram S. Eedara R. V. V. Yadav S. Rabishankar O. (2018). *Planctopirus hydrillae* sp. nov., an antibiotic producing *Planctomycete* isolated from the aquatic plant *Hydrilla* and its whole genome shotgun sequence analysis. *J. Antibiot.* 71 575–583. 10.1038/s41429-018-0035-1 29467380

