# Supplementary material for: Stieleriacines, N-Acyl Dehydrotyrosines From the Marine Planctomycete Stieleria neptunia sp. nov
Source: Front Microbiol. 2020 Jul 16;11:1408. doi: 10.3389/fmicb.2020.01408 (PMC7378531; doi:10.3389/fmicb.2020.01408)
Supplement: Supplementary file 1 [file Data_Sheet_1.pdf]

Supplementary Material

Article

Stieleriacines, *N*-Acyl Dehydrotyrosines from the Marine Planctomycete *Stieleria neptunia* sp. nov.

Birthe Sandargo[1,4,†], Olga Jeske[2,†], Christian Boedeker[2], Sandra Wiegand[3], Jan-Peer Wennrich[1,4], Nicolai Kallscheuer[3], Mareike Jogler[5], Manfred Rohde[6], Christian Jogler[3,5]* and Frank Surup[1,4,]*

[1] Microbial Drugs Department, Helmholtz-Centre for Infection Research (HZI), Inhoffenstr. 7, 38124 Braunschweig, Germany; birthe.sandargo@helmholtz-hzi.de, jan-peer.wennrich@helmholtz-hzi.de, frank.surup@helmholtz-hzi.de.

[2] Leibniz Institute DSMZ - Deutsche Sammlung von Mikroorganismen und Zellkulturen, Inhoffenstr. 7b, 38124 Braunschweig, Germany; oje12@dsmz.de; c.boedeker.dsmz@gmail.com;

[3] Department of Microbiology, Radboud University, Heyendaalseweg 135, 6525 AJ Nijmegen, Netherlands; s.wiegand@science.ru.nl; n.kallscheuer@science.ru.nl;

[4] German Centre for Infection Research (DZIF), partner site Hannover-Braunschweig, 38124 Braunschweig, Germany.

[5] Friedrich-Schiller-University Jena, Institute of Microbiology, Department of Microbial Interactions, 07743 Jena, Germany; mareike@jogler.de; christian@jogler.de

[6] Central Facility for Microscopy, Helmholtz-Centre for Infection Research (HZI), Inhoffenstr. 7, 38124 Braunschweig, Germany; manfred.rohde@helmholtz-hzi.de.

[†] these authors contributed equally to this work

***** Correspondence: christian@jogler.de (biology), frank.surup@helmholtz-hzi.de (chemistry); Tel.: +49-351-6181-4256

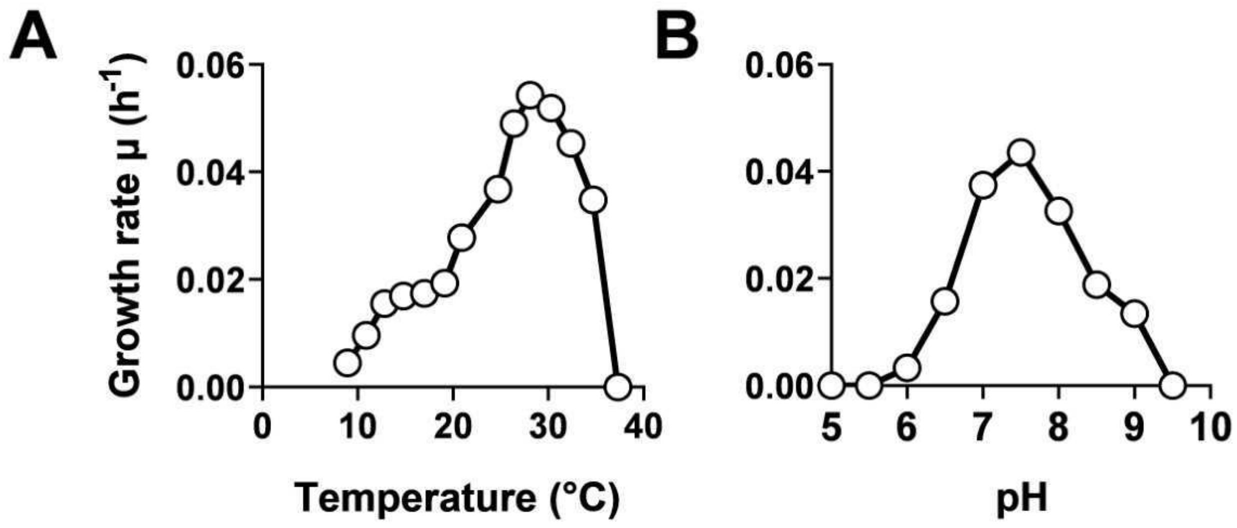

Figure S1: Temperature and pH optimum of strain Enr13^T. **A**) For determination of the optimal growth temperature, optical density measured at 600 nm (OD_{600}), was followed for a cultivation time of 160 hours. The calculated growth rates for the different tested cultivation temperatures are shown. Data represent mean values from triplicates. All cultivations were performed at pH 8.0. **B**) For determination of the optimal pH OD_{600} was followed for a cultivation time of 160 hours. Calculated growth rates for the different tested pH values are shown. Data represent mean values from triplicate measurements. All cultivations were performed at 28 °C.

Table S1: Genome information of strain Enr13^T and its current closest relatives.

	Enr13^T	*Stieleria maiorica* Mal15^T	*Rubripirellula obstinata* LF1^T	*Rhodopirellula baltica* SH 1^T	*Roseimaritima ulvae* UC8^T
Genome size (bp)	10,975,817	9,894,293	7,094,218	7,145,576	8,212,515
Scaffolds	1	1	27	1	1
N50 (scaffolds)	10,975,817	9,894,293	6,586,311	7,145,576	8,212,515
Completeness (%)	98.3	98.3	98.3	98.3	98.3
G+C content (%)	58.9	59.3	54.3 (± 1.7)	55.4	59.1
Coding density (%)	86.0	87.0	84.4	88.7	87.5
Total genes	7,904	7,016	5,950	5,569	5,919
Genes/Mb	720.1	709.1	838.7	779.4	720.7
Protein-coding genes	7,797	6,920	5,870	5,465	5,815
Protein-coding genes/Mb	710	700	827	765	708
Hypothetical proteins (%)	43.9	41.9	49.2	56.3	39.5
tRNA genes	99	81	67	91	71
tRNA genes/Mb	9.0	8.2	9.4	12.7	8.6
16S rRNA genes	3	3	2	1	1
Transposable elements	54	17	72	0	6
Transposable elements/Mb	4.9	1.7	10.1	0.0	0.7
Giant genes	14	13	9	6	10

Table S2: Phylogenetic comparisons between strain Enr13^T and its current closest relatives.

	16S rRNA identity (%)	*rpoB* identity (%)	Average nucleotide identity (%)
**Enr13^T**	100	100	100
Stieleria maiorica Mal15^T	99.5	97.6	80.1
Rubripirellula obstinata LF1^T	93.4	87.4	70.3
Rhodopirellula baltica SH 1^T	94.3	86.7	69.8
Roseimaritima ulvae UC8^T	93.2	85.1	70.5

Table S3: Minimum inhibitory concentration (MIC) for bacteria, yeasts and fungi in µg/mL. MeOH (20 µL) was used as negative control and displayed no inhibitory effects. The following reference antimicrobials were used: [O] Oxytetracycline, [G] Gentamycin, [K] Kanamycin for bacteria; [N] Nystatin for yeasts and fungi; n. i. = no inhibition up to 100 µg/mL.

Test organism	Strain number	O-methyl-Stieleriacine	Reference [N, O, G, K]
Schizosaccharomyces pombe	DSM70572	n.i.	33.3 [N]
Pichia anomala	DSM6766	n.i.	66.7 [N]
Mucor hiemalis	DSM2656	n.i.	33.3 [N]
Candida albicans	DSM1665	n.i.	66.7 [N]
Rhodoturula glutinis	DSM10134	n.i.	16.7 [N]
Micrococcus luteus	DSM1790	16.7	0.8 [O]
Bacillus subtilis	DSM10	n.i.	16.7 [O]
Escherichia coli	DSM1116	n.i.	3.3 [O]
Staphylococcus aureus	DSM346	66.7	0.4 [O]
Mycobacterium smegmatis	ATCC700084	n.i.	3.3 [K]
Chromobacterium violaceum	DSM30191	n.i.	0.8 [O]
Pseudomonas aeruginosa	PA14	n.i.	0.8 [G]

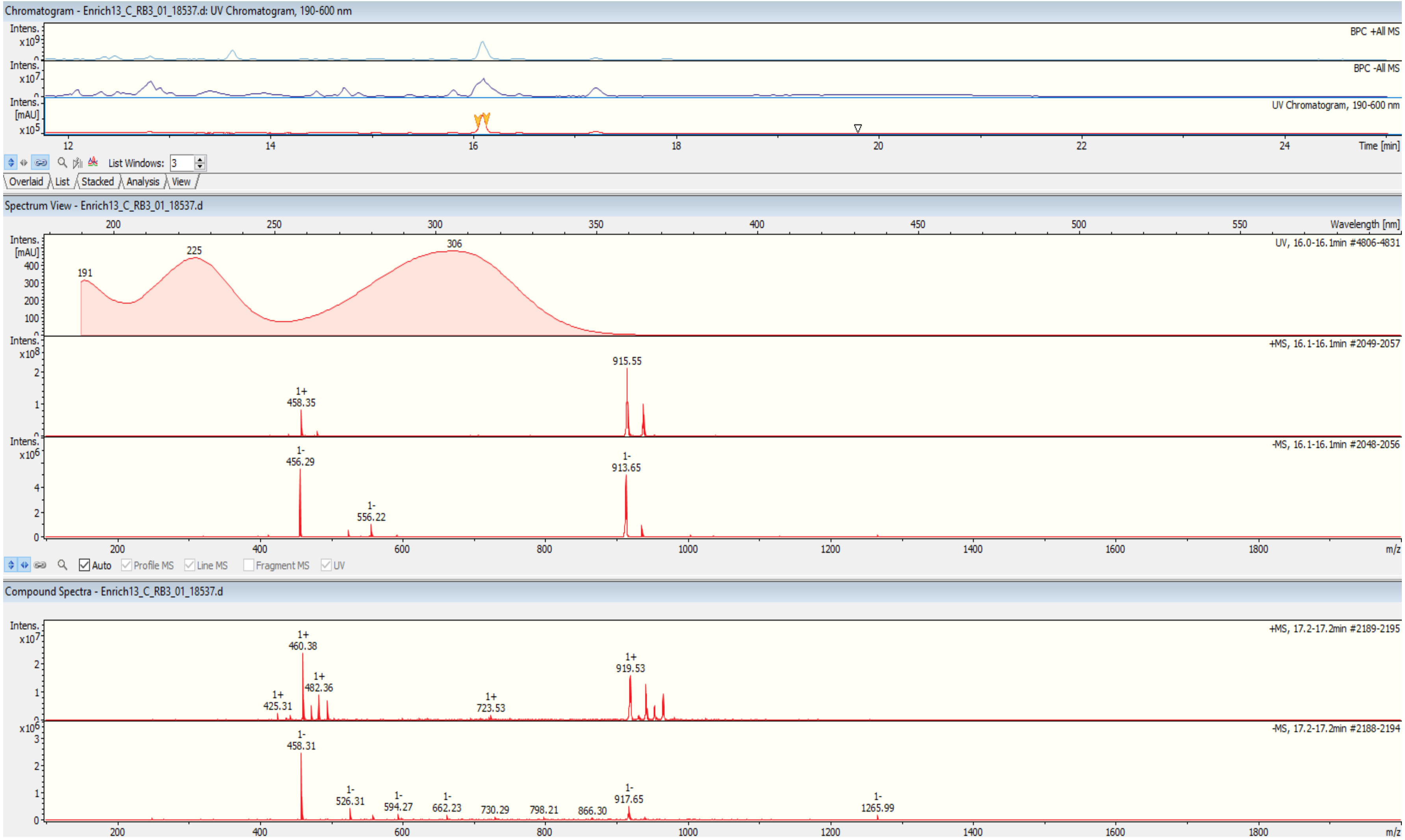

Figure S3: ESI-MS spectrum of **1**.

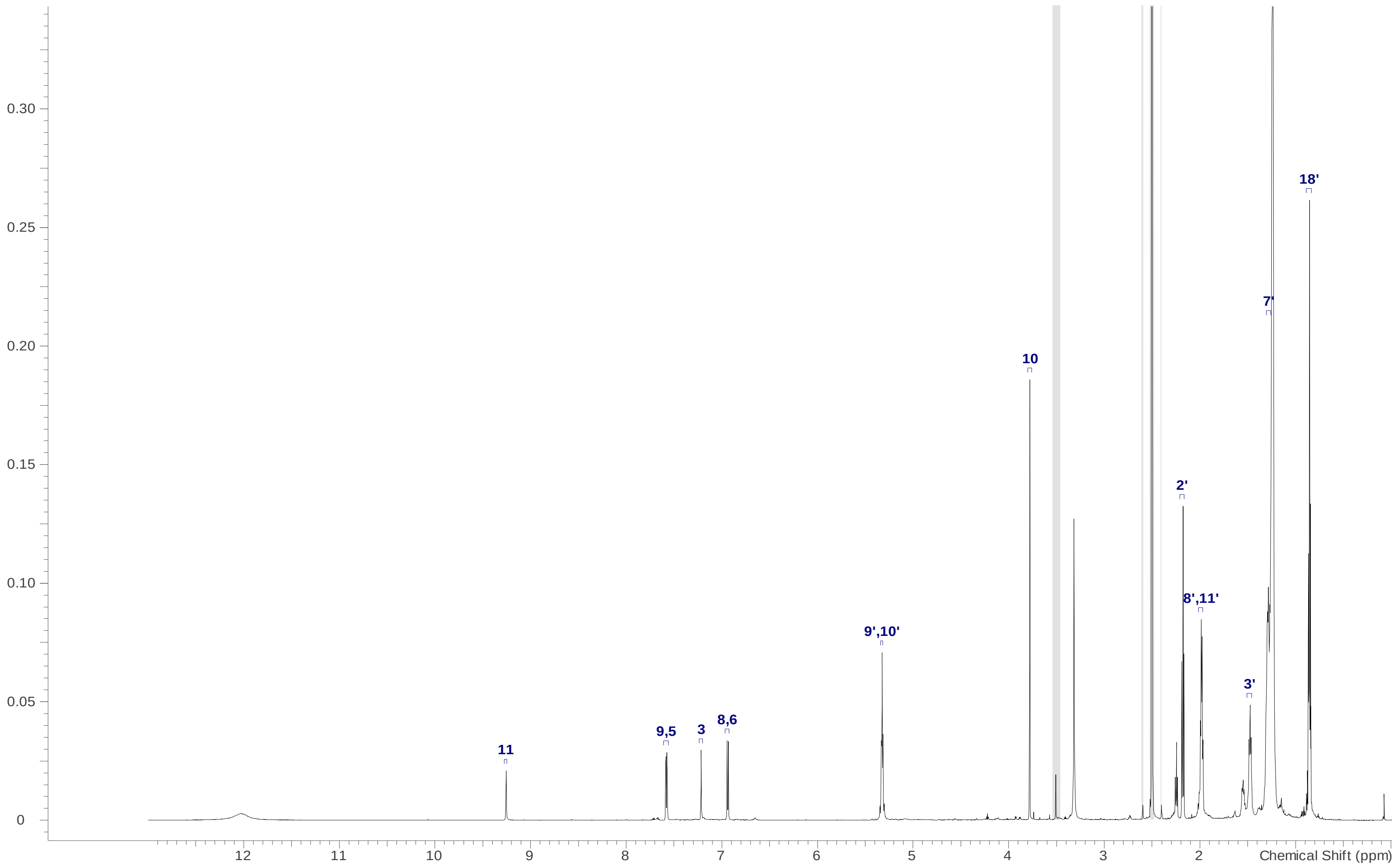

Figure S4: ^{1}H NMR spectral data (700 MHz, DMSO-d_6) of **1**.

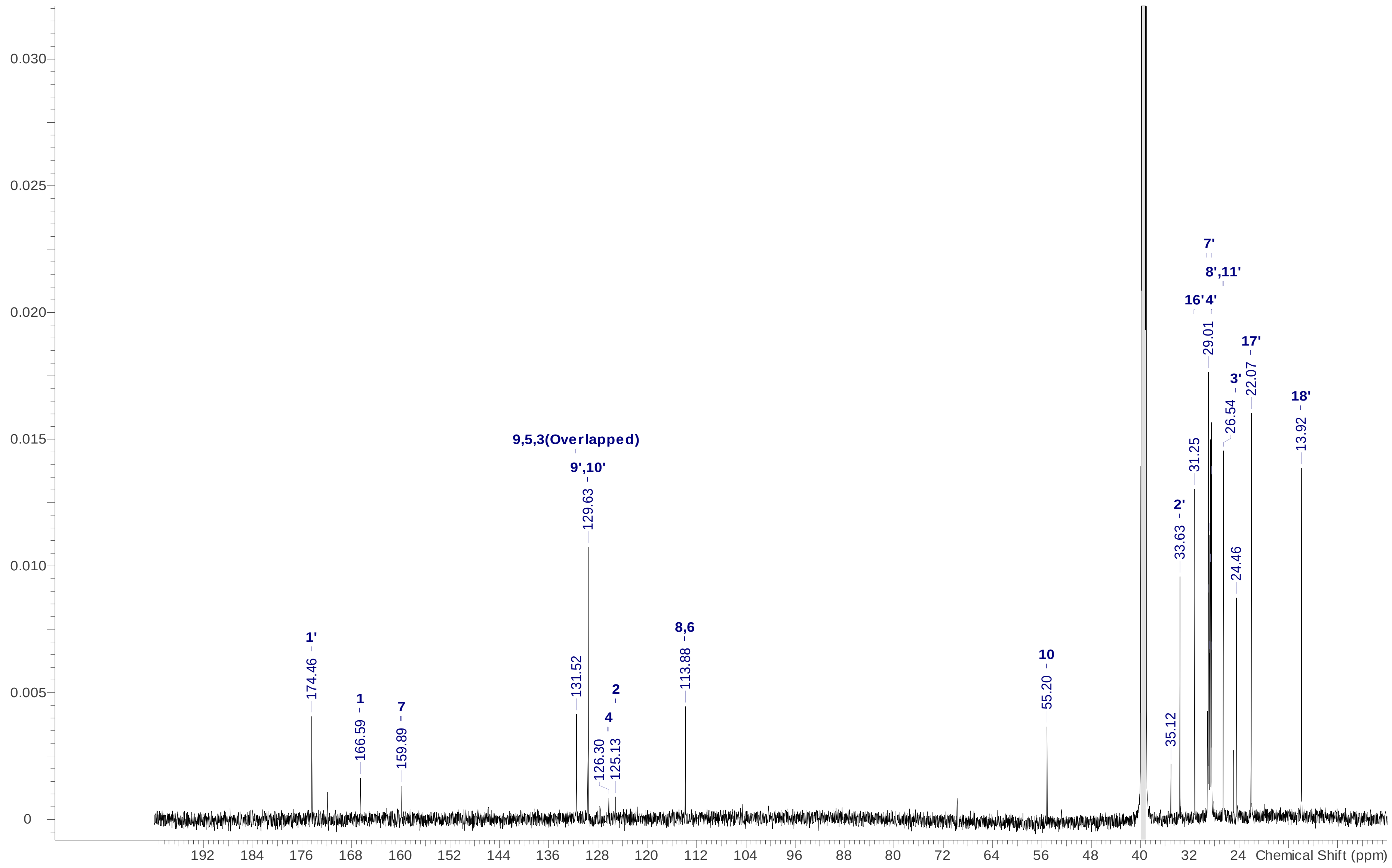

Figure S5: NMR spectral data (175 MHz, DMSO-d_6) of **1**

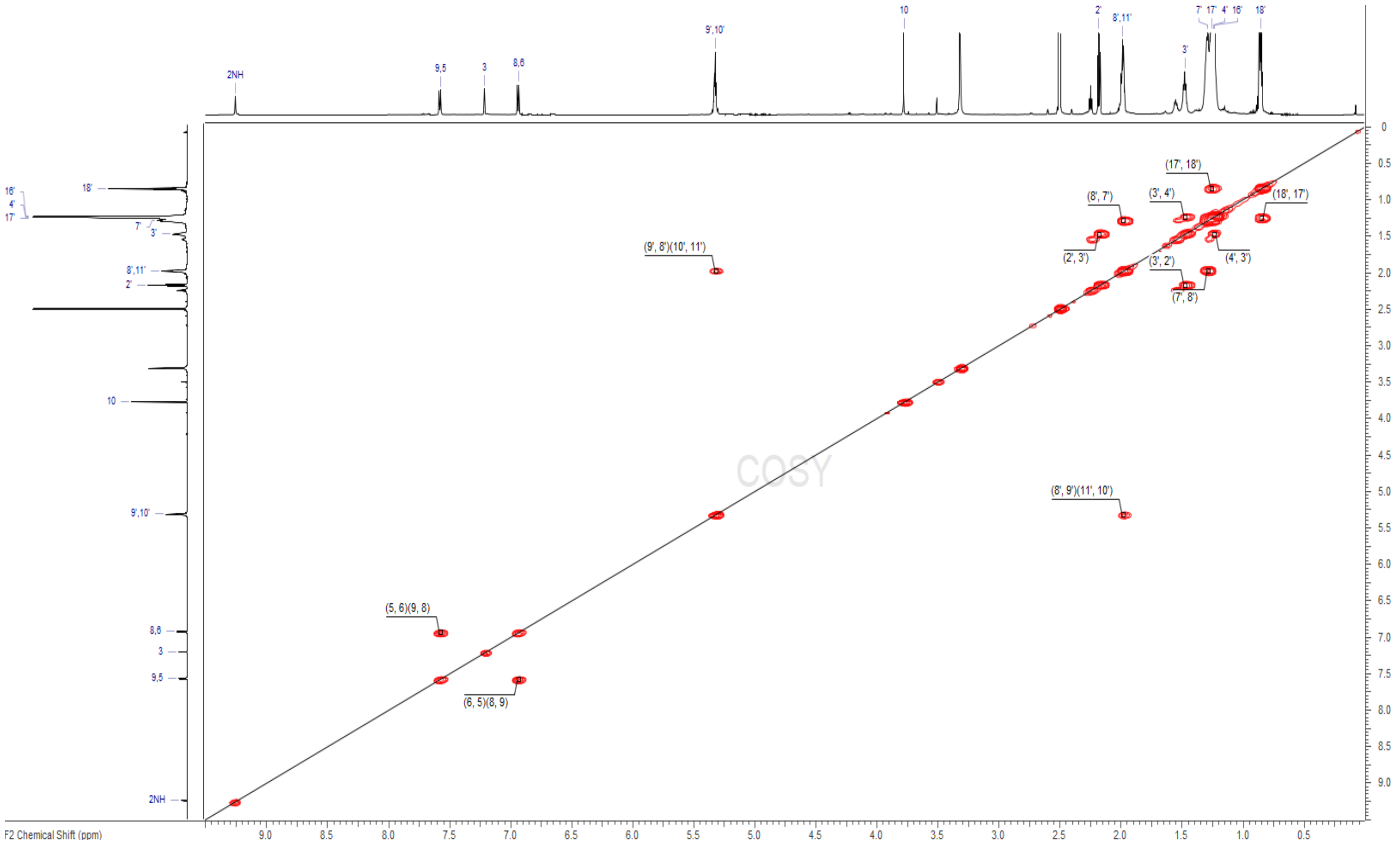

Figure S6: COSY NMR spectral data (700 MHz, DMSO-d_6) of **1**.

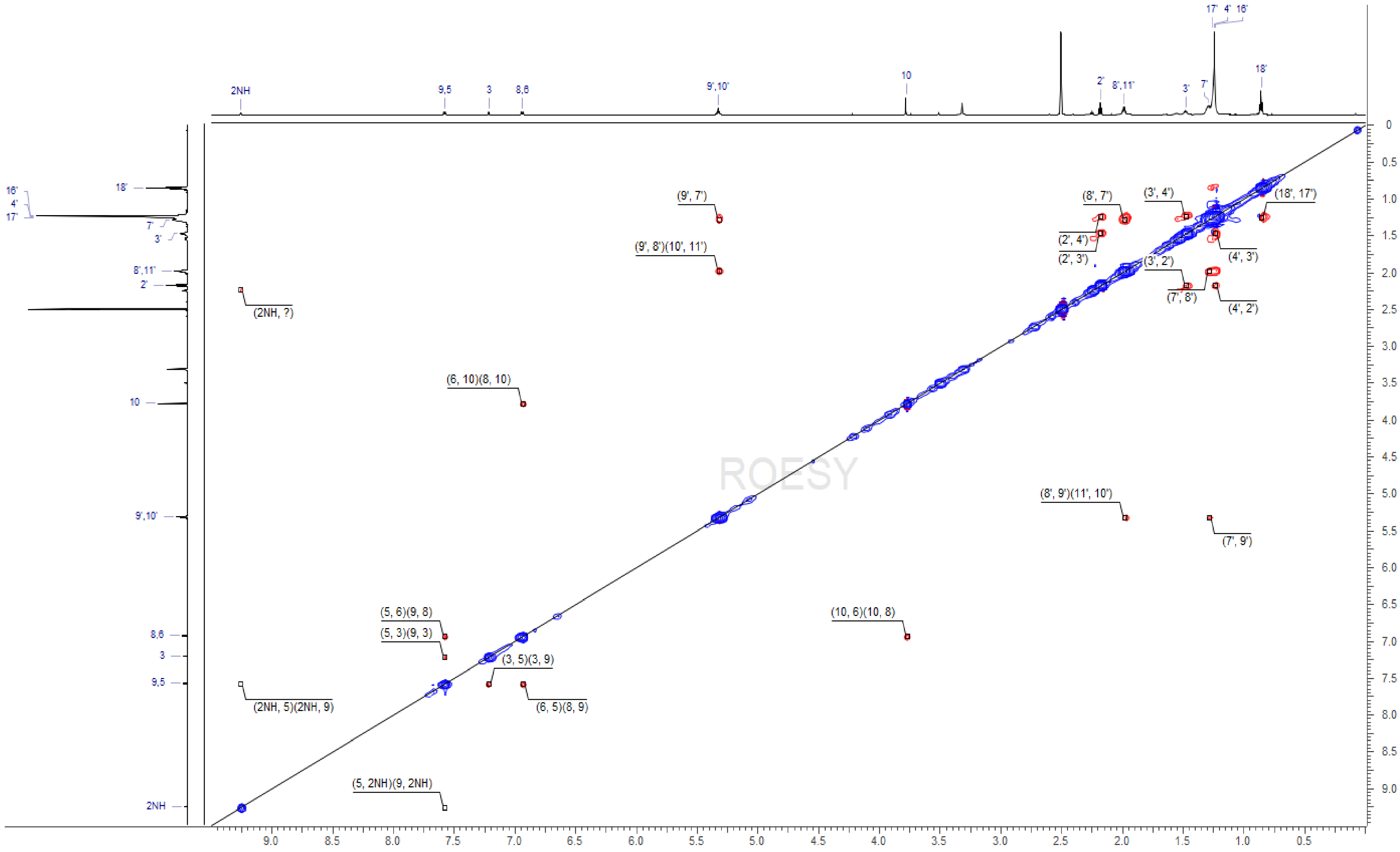

Figure S7: ROESY NMR spectral data (700 MHz, DMSO-d_6) of **1**.

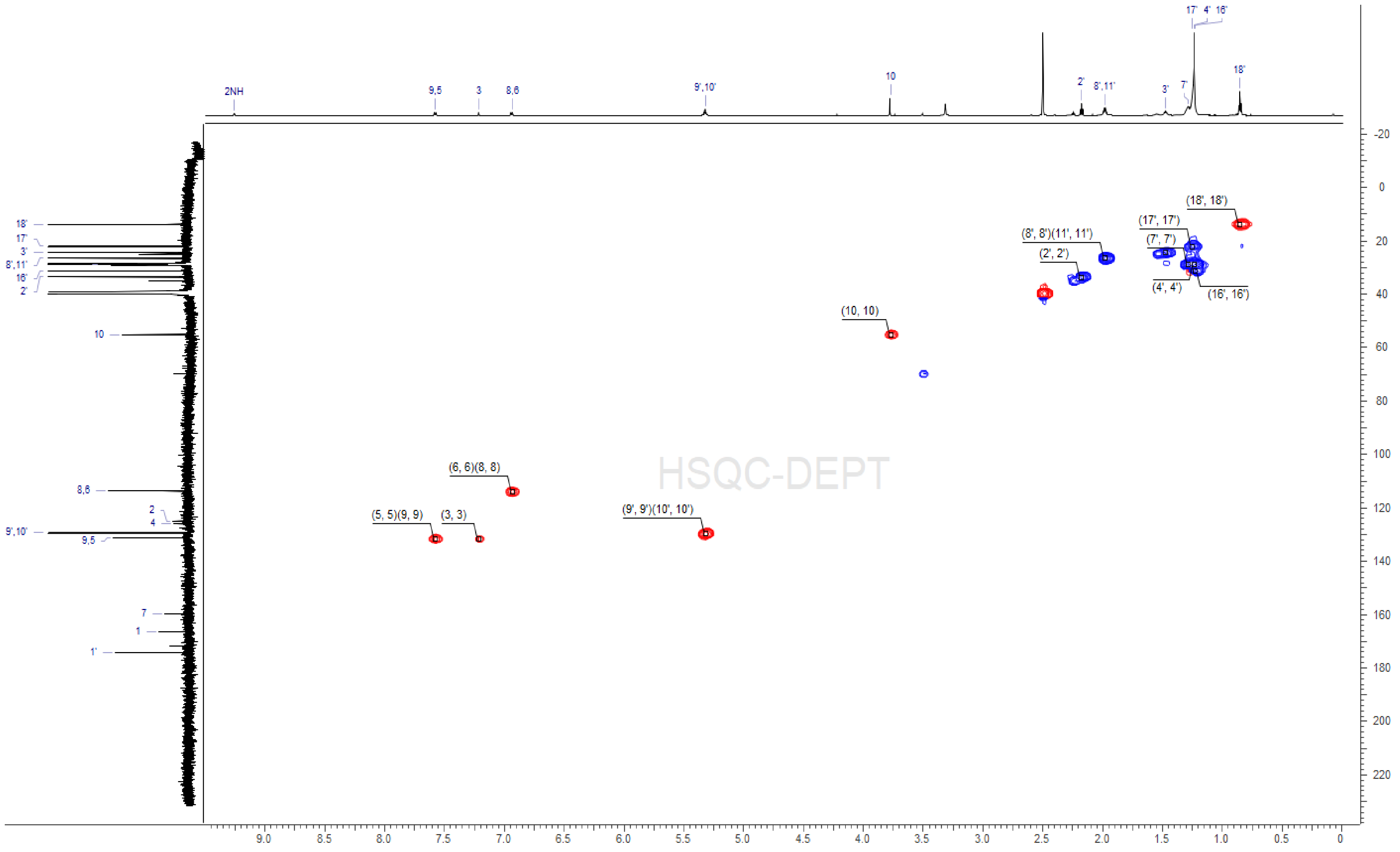

Figure S8: HSQC NMR spectral data (700 MHz, DMSO-d_6) of **1**.

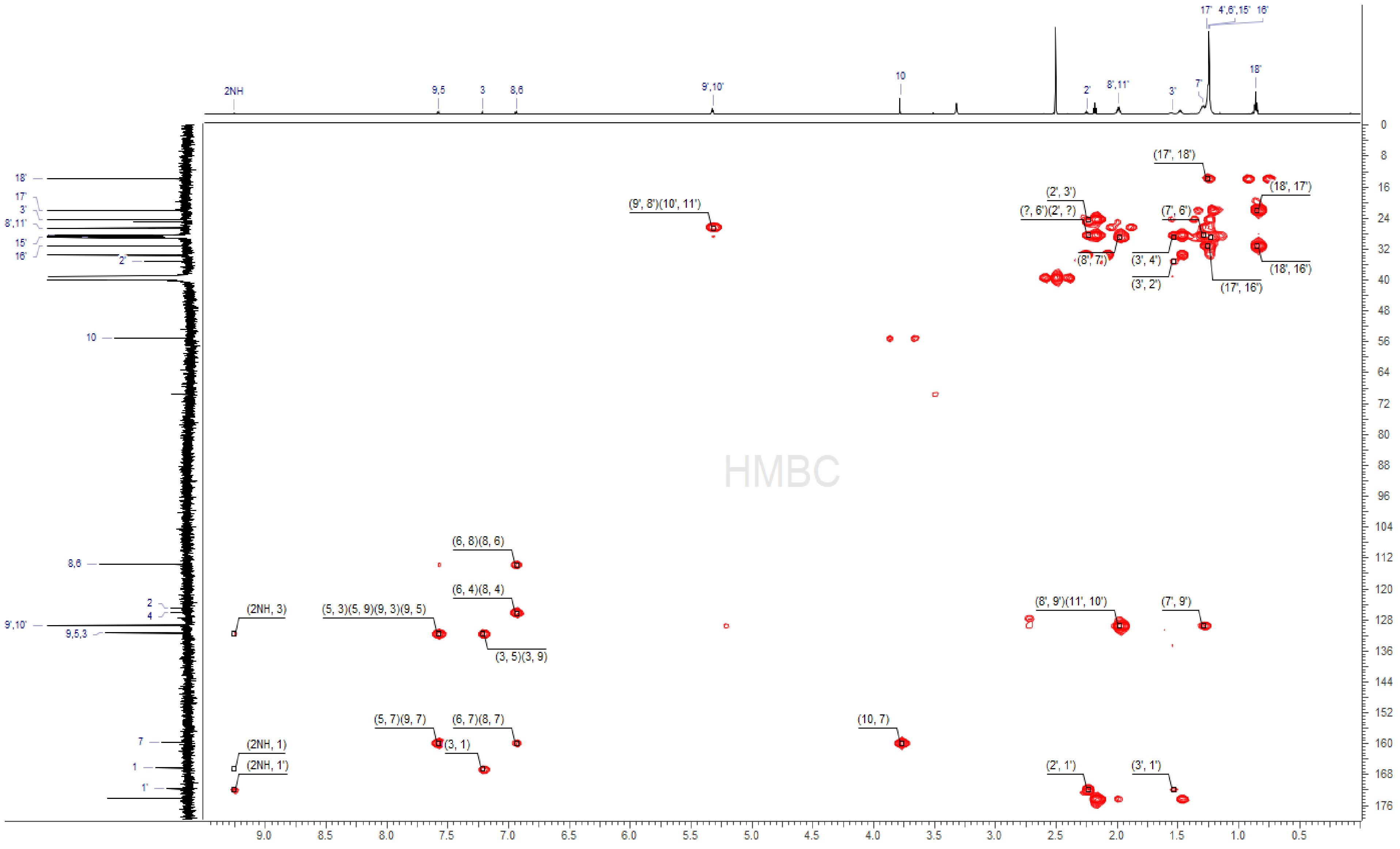

Figure S9: HMBC NMR spectral data (700 MHz, DMSO-d_6) of **1**.

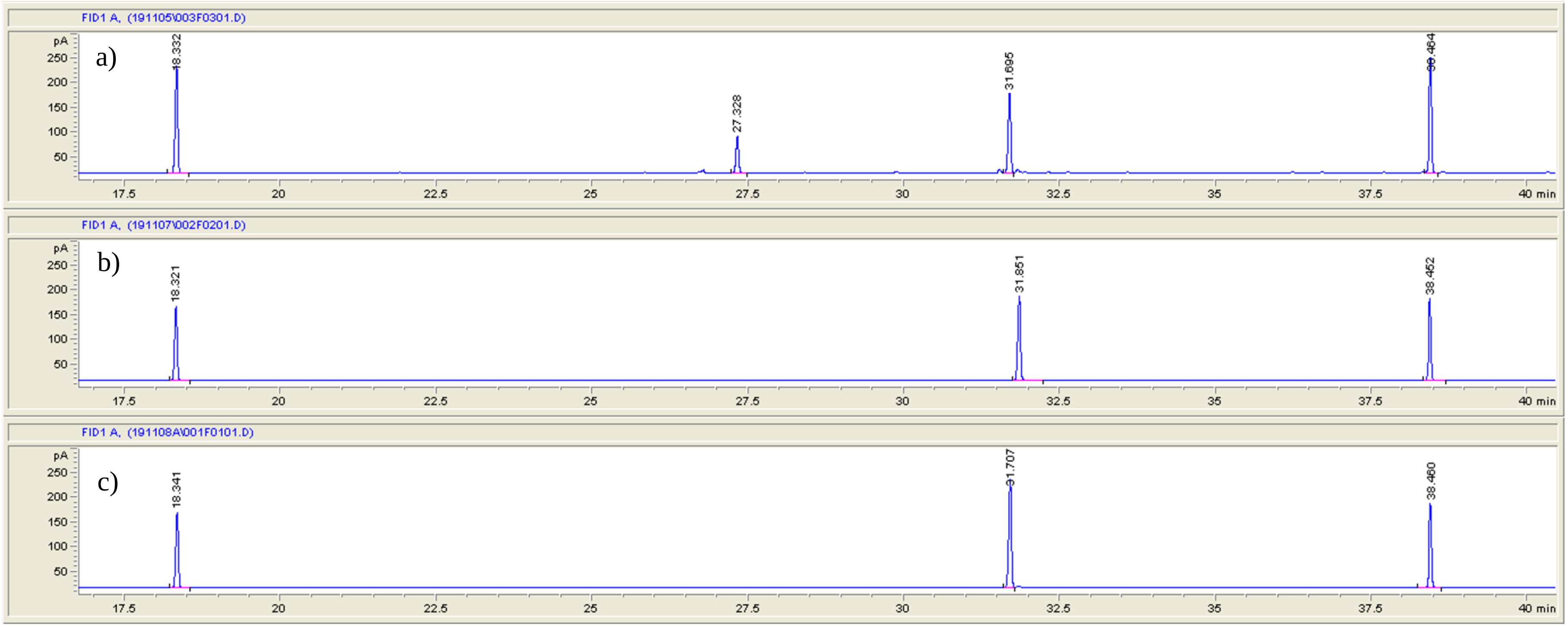

Figure S10: Fatty acid methyl ester analysis. a) Sample obtained from **1**, **b**) authentic oleic acid standard, **c**) authentic elaidic acid standard. Retention times of authentic standards in GC-MS database: t_R 32.117 = 18:1ω5, t_R 31.107 = 18:1ω6c, t_R 31.78 = 18:1ω6t, t_R 31.985 = 18:1ω7t (*trans*-vaccenic acid), t_R 31.873 = 18:1ω7c (*cis*-vaccenic acid), t_R 31.704 = 18:1ω9c (oleic acid), t_R 31.733 = 18:1ω9t (elaidic acid), t_R 31.733 = 18:1ω12c. Peaks at t_R = 18.332 and t_R = 38.464 are hexadecane and tetracosan standards, respectively.
